# Has the prevalence of Chagas disease increased in Central Latin America?

**DOI:** 10.1371/journal.pntd.0008851

**Published:** 2020-12-03

**Authors:** Kota Yoshioka, Ken Hashimoto, Carlota Monroy

**Affiliations:** 1 Institute of Tropical Medicine, Nagasaki University, Japan; 2 Independent Global Health Consultant. Kakogawa, Japan; 3 Facultad de Ciencias Químicas y Farmacia, Escuela de Biología, LENAP, Universidad de San Carlos de Guatemala, Guatemala City, Guatemala; US Food and Drug Administration, UNITED STATES

In their editorial published in PLOS NTDs, Hotez et al. [1] wrote that “the GBD 2017 [Global Burden of Disease Study 2017] tells a sobering story about progress in controlling malaria and NTDs [neglected tropical diseases]” in Central Latin America (CLA). CLA is a region defined by the GBD 2017, including Mexico, Guatemala, El Salvador, Honduras, Nicaragua, Costa Rica, Panama, Colombia and Venezuela. As part of the sobering story, Hotez et al. [1] found that the prevalence of Chagas disease increased by 16% from 2000 to 2017, implying that its control has not progressed during the last two decades. In this article, we show that the GBD 2017 can tell a different story about Chagas disease in CLA.

Among a number of estimates provided by the GBD 2017, Hotez et al. chose prevalence expressed in numbers (i.e. prevalent cases or absolute number of infected cases), as a single indicator to assess progress in the control of Chagas disease. They found that the estimated numbers of cases rose from 1.33 million in 2000 to 1.54 million in 2017 in CLA. Absolute numbers of cases are useful to show the size of a health problem in a defined population at a given point in time. But they are generally not useful for comparisons over decades because populations may change in size and age structure. Since the population in CLA grew by nearly 10% between 2000 and 2017 according to the GDB 2017 [2], comparison of absolute numbers can be misleading.

Instead of absolute numbers of infected cases, we chose and compared age-standardized prevalence of Chagas disease, expressed in percentage, extracted from the same GDB 2017 database [3]. Age-standardized prevalence is more suitably comparable than absolute numbers, when age structure of a population differs between two time points and prevalence varies by age. CLA has been in demographic transition: for example, the proportion of the population aged 0 to 14 years dropped from 35% in 2000 to 27% in 2017 [4]. The prevalence of Chagas disease is known to be higher among older populations [5,6]. In short, the proportion of young low-prevalent population has shrunk in this region over time, which justifies our choice of age-standardized prevalence.

**Fig 1:**
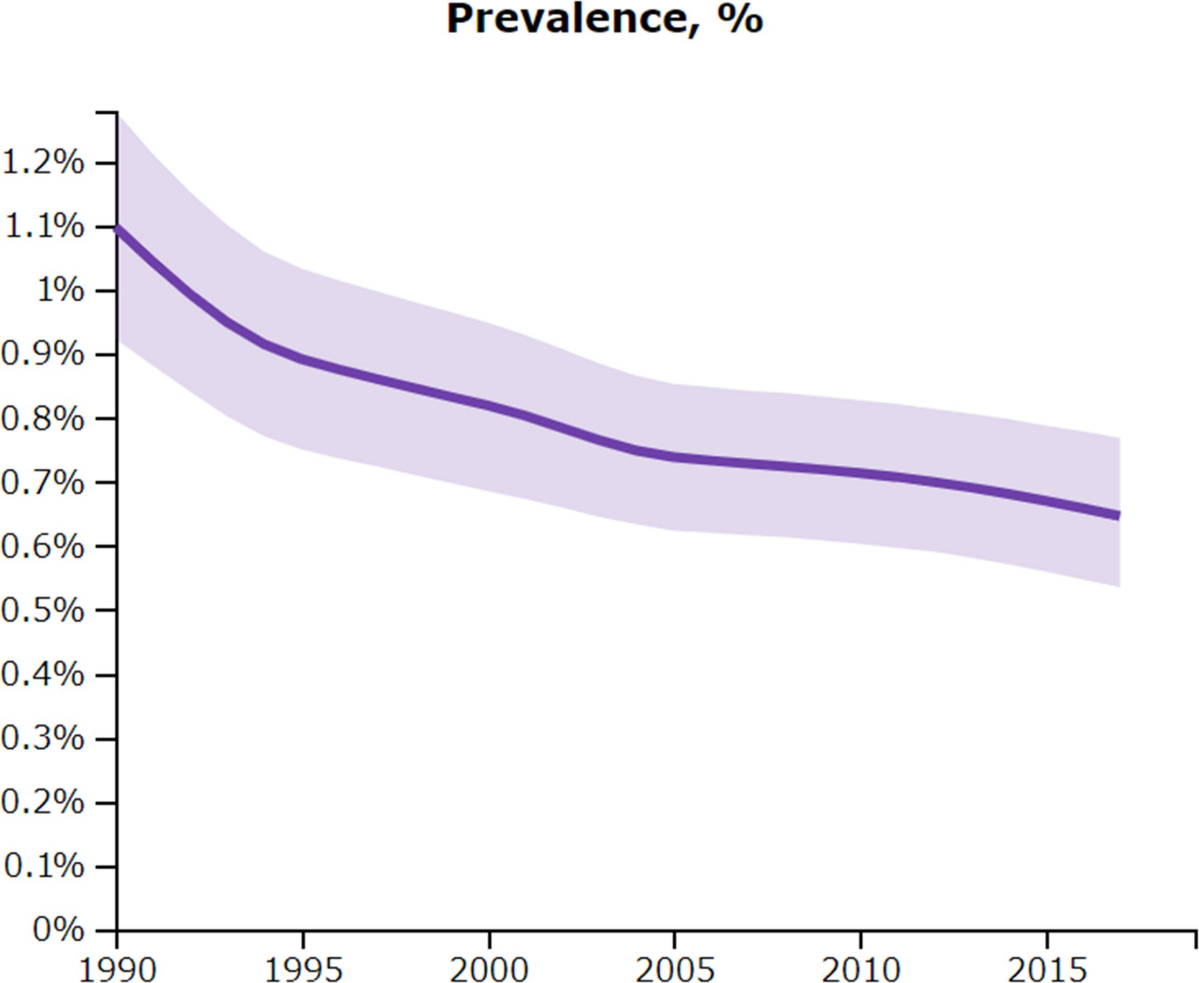
Age-standardized prevalence of Chagas disease in Central Latin America, 1990–2017. The light purple band represents 95% uncertainty intervals. Source: GBD 2017 [3]. Permalink http://ghdx.healthdata.org/gbd-results-tool?params=gbd-api-2017-permalink/5e2d83a01cecfb521ebed961813e43b6

Fig 1 shows the longitudinal trend of age-standardized prevalence of Chagas disease in CLA. Once controlled for the effects of population size and age, the prevalence steadily declines in the last three decades or so. Between 2000 and 2017, the period examined by Hotez et al., the age-standardized prevalence decreased from 0.82% to 0.65%, which is a 21% decline. This declining trend can be interpreted as showing a promising story about progress in Chagas disease control, in contrary to the picture presented by Hotez et al.

Our view of the promising story can be corroborated by remarkable successes in controlling Chagas disease in CLA during the past two decades. For example, nearly 100% of blood products are currently screened to prevent transfusional transmission of *Trypanosoma cruzi*, the causal agent of Chagas disease. In 2006, only El Salvador and Costa Rica reported universal screening for *T*. *cruzi* [7]. By 2009, all CLA countries, except for Mexico and Venezuela, achieved 100% screening [7]. By 2014, Mexico and Venezuela reached 96% and 100% screening coverage, respectively [8]. Another example is elimination of *Rhodnius prolixus*, the vector that was far more effective in transmitting *T*. *cruzi* than local secondary vectors [5], and therefore, was responsible for most of the endemicity of Chagas disease in Central America and Mexico [9]. Organized control efforts against *R*. *prolixus* started in 1997 and interruption of *T*. *cruzi* transmission by *R*. *prolixus* was certified by Pan American Health Organization in Guatemala, Honduras and Nicaragua by 2011 [10,11]. Central America and Mexico are now free of *T*. *cruzi* transmission by *R*. *prolixus* [9]. As a result of these achievements, it is logical that incidence of *T*. *cruzi* transmission has decreased substantially in CLA.

**Fig 2:**
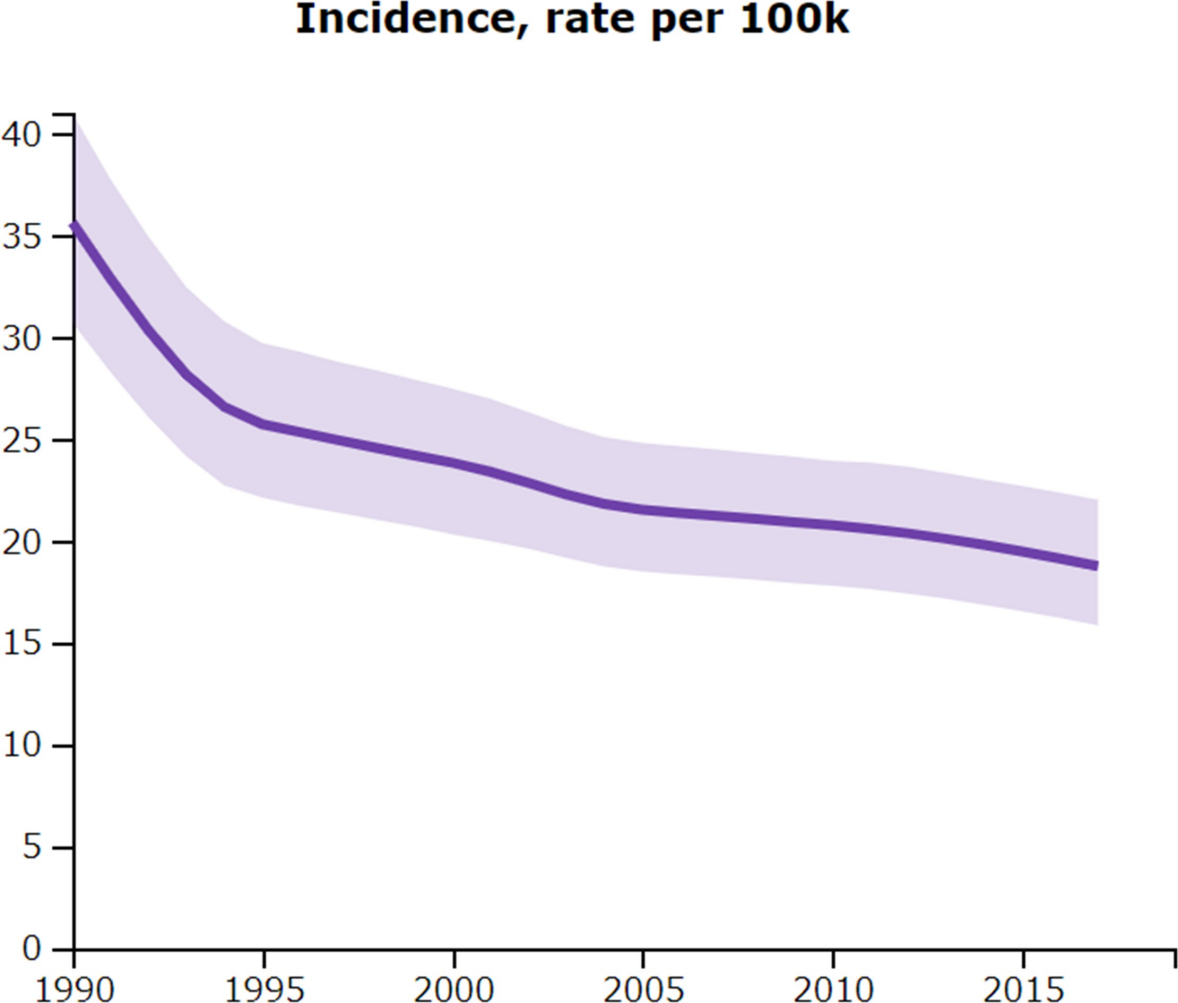
Age-standardized incidence of Chagas disease in Central Latin America, 1990–2017. The light purple band represents 95% uncertainty intervals. Source: GBD 2017 [3]. Permalink http://ghdx.healthdata.org/gbd-results-tool?params=gbd-api-2017-permalink/5e2d83a01cecfb521ebed961813e43b6

Nevertheless, the impact of the above-mentioned control efforts is not clear in the GBD 2017 estimates. Fig 2 shows that the overall trend of age-standardized incidence steadily declined from 2000 to 2017, but this decline is not statistically significant (which can be observed as the overlapping 95% uncertainty intervals between 2000 [20.32–27.46] and 2017 [15.84–22.02] [3]). We believe that the impact of the control efforts is not adequately captured by the model used for calculation of the GBD 2017 estimates. One recommendation for the future GBD study is to incorporate correction into the modelling procedure, a similar step taken by the GBD 2017 to consider interruption of vector-borne transmission in Chile and Uruguay (see James et al. [12]: Supplementary appendix 1, p.106-107). Such a correction for CLA should also capture continuing *T*. *cruzi* transmission by vector species other than *R*. *prolixus* as well as vertical transmission. In addition, regular epidemiological surveys and reporting from national health information systems, both of which are currently lacking, will improve the GBD estimates.

As shown above, we found that the relative change in prevalence between 2000 and 2017 dropped from 16% to -21%, after adjusting for population size and age. This finding implies that the rise of prevalent cases is driven largely by changes in population size and age structure in CLA, therefore, weakens some of the arguments made by Hotez et al. For instance, their editorial tries to link the rise of Chagas disease prevalent cases in the Northern Triangle (Guatemala, El Salvador and Honduras) to intensified violence and other political and economic instability. Although the political and other contextual factors may play some roles in influencing the numbers of prevalent cases, it does not seem to be appropriate to reason the increase in prevalent cases without considering the role of demographic changes.

In this article, we argued that the story about progress in controlling Chagas disease in CLA is rather promising than sobering. We appreciate Hotez et al.’s call to pay more attention to the links among infectious disease control and political, economic and social contexts in CLA. However, our analysis showed that CLA has gained important achievements in Chagas disease control despite this region’s several vulnerabilities. Our article also suggests that progress of disease control should not be assessed by a single indicator and that there is a need to improve GBD estimates about Chagas disease in CLA. We believe that CLA countries have much potential for controlling NTDs and malaria, with sound understanding of the region’s context and support from scientific and international communities.
